# Structural Analysis and Conformational Dynamics of Short Helical Hyperphosphorylated Segments of Tau Protein (Sequence 254–290) in Alzheimer’s Disease: A Molecular Dynamics Simulation Study

**DOI:** 10.3389/fmolb.2022.884705

**Published:** 2022-08-08

**Authors:** Mozhgan Alipour, Mahsa Motavaf, Parviz Abdolmaleki, Alireza Zali, Farzad Ashrafi, Saeid Safari, Behnam Hajipour-Verdom

**Affiliations:** ^1^ Functional Neurosurgery Research Center, Shohada Tajrish Comprehensive Neurosurgical Center of Excellence, Shahid Beheshti University of Medical Sciences, Tehran, Iran; ^2^ Department of Biophysics, Faculty of Biological Sciences, Tarbiat Modares University, Tehran, Iran

**Keywords:** Alzheimer’s disease, tau protein, hyperphosphorylation, conformational dynamics, molecular dynamics simulation

## Abstract

Alzheimer’s disease (AD) is a progressive neurodegenerative disorder whose early diagnosis leads to a chance for successful treatment and decreases the side effects. Hyperphosphorylation of tau proteins is a pathological hallmark of AD that causes it to lose its attachment ability to the microtubules. Alteration of tau structure due to its hyperphosphorylation is an exciting challenge regarding AD treatments. Here, we aimed to examine the structural alterations of short helical segments of tau protein with one to three phosphorylated sites by molecular dynamics simulation. Results indicated that the interaction of two similar segments with three phosphorylated sites (P-Ser262, 285, and 289) formed a compact and more stable structure than the one phosphorylated site complex (P-Ser262). Moreover, due to the high dynamics of the P-Ser262 complex, several structures were made with different conformational dynamics, but there was only one stable cluster of the P-Ser262, 285, and 289 complex during simulation. It seems that the P-Ser262, 285, and 289 complex plays an important role in the formation of paired helical filaments (PHFs) by forming a stable dimer. Generally, it is important to identify how structural features of segments in tau protein change when the phosphorylated sites increase from one to three sites and their effects on the formation of PHFs for drug design and diagnostic biomarkers.

## Introduction

Alzheimer’s disease (AD) is a neuropathological disease characterized by two lesions, including the formation of amyloid plaques and abnormally phosphorylated tau proteins. So far, more than 20 different neurological diseases have been identified in humans that are associated with the abnormal accumulation of tau protein (Williams, 2006; Iqbal et al., 2010). This protein exists in six isoforms in the human brain and is expressed by a gene on chromosome 17. They vary in length from 352 to 441 amino acids created by different pre-mRNA splicing and post-translational modifications (Nizynski et al., 2017; Dzwolak et al., 2004; Wang and Mandelkow, 2016). Moreover, each isoform comprises three main domains, including the projection domains, the proline-rich domain, and the microtubule-binding domains (MBDs). The projection domain is made of acidic residues followed by a proline-rich region, and the MBD consists of several repeats of microtubule-binding sequences that consist of 31 or 32 residues (Sottejeau et al., 2015; Barbier et al., 2019).

The interaction between filamentous actin (F-actin) and microtubules is important to stabilize the cytoskeleton, which has an essential role in cellular processes such as axonal growth, cell division, migration, and development of neurons. The interaction is mediated through the tau protein. Tau binds simultaneously to microtubules and actin filaments (Fulga et al., 2007; Mohan and John, 2015). Studies have shown that tau is bonded to F-actin with high affinity by repeated domains of tau protein and that residues 254–290 have an essential role in the interactions. The segment (254–290) has two important motifs, including KXGS and VQIINK, which exhibited high preference values for phosphorylation and aggregation (Von Bergen et al., 2000; Cabrales Fontela et al., 2017). Moreover, the VQIINK motif is important for forming paired helical filaments (PHFs). Phosphorylation at serine 262 attenuates the binding of tau protein to F-actin but alone cannot lead to the formation of PHFs (Seubert et al., 1995; Schneider et al., 1999a).

When the tau protein has malfunctioned, it causes a wide range of problems such as progressive supranuclear palsy (PSP), corticobasal degeneration (CBD), traumatic brain injury (TBI), and frontotemporal dementia (FTD) (Graff-Radford and Woodruff, 2007; Mietelska-Porowska et al., 2014). Studies have shown that the amount of soluble tau protein in the brain of an Alzheimer’s patient is the same as in the brain of a healthy person, but the phosphorylated sites per tau molecule are about four times higher than those in a healthy person. There are naturally 2–3 moles of phosphorylated tau protein in the brain of a healthy person (Iqbal et al., 2010; Wang et al., 2016; Naseri et al., 2019).

When the phosphorylation of tau proteins increases abnormally (hyperphosphorylation), it leads to the polymerization of these proteins, which form neurofibrillary tangles (NFTs) and neuropil threads (NTs) (Omalu et al., 2010; Pradeepkiran et al., 2019). More than 85 sites in the tau protein are known to have the potential to be phosphorylated, including 5 tyrosine, 35 threonine, and 45 serine residues. Due to the expanded structure of tau, protein kinases can easily access their target sites for phosphorylation, so the amount of phosphorylation in this protein is highly variable. To date, no study has shown that all of these sites in the protein are phosphorylated simultaneously (Martin et al., 2011; Perez et al., 2011; Neddens et al., 2018).

Phosphorylation at all of these sites is not entirely independent, meaning that phosphorylation at one site affects phosphorylation at another site. Many kinases are involved in the phosphorylation of tau proteins such as PKA, CaMKII, PKC, and MAPKs (Kimura et al., 2018; Ramkumar et al., 2018). Tau phosphorylation is performed on specific motifs such as KXGS, so kinases identify those. Phosphorylated Ser262 within the KXGS motifs is an important factor in characterizing AD (Schwalbe et al., 2013; Cook et al., 2014; Kellogg et al., 2018). Increased phosphorylation of tau protein leads to the formation of PHF that hexapeptide VQIINK (PHF6*) and VQIVYK (PHF6) play an essential role in this formation. Ser285, Ser289, Ser293, Ser305, and Tyr310, located near the hexapeptides, are identified residues for phosphorylation (Hanger et al., 2007; Smit et al., 2017; Liu et al., 2019; Pradeepkiran et al., 2022). On the other hand, Ser285 and Ser289 near the C-terminus of the PHF6* and PHF6 sequences are known to be phosphorylation hotspots for casein kinase 1 (CK1), casein kinase 2 (CK2), and glycogen synthase kinase-3 (GSK-3) in the brains of Alzheimer’s disease patients. Also, phosphorylation at these sites leads to the formation of PHFs (Mendoza et al., 2013; Pradeepkiran and Reddy, 2019). However, the mechanism of its formation is not clearly understood. One of the most critical challenges in designing a biomarker for the early detection of AD is how the structure of a particular region of tau protein changes as the number of phosphorylated sites increases.

The present study aimed to identify how structural features of two docked similar segments of tau protein change when the phosphorylated sites increase from one site in Ser262 to three sites in Ser262, Ser285, and Ser289 and their effects on PHF formation. Studies have shown that phosphorylation in Ser262 decreases the binding of tau to microtubules and plays a critical role in tau accumulation and toxicity (Biernat et al., 1993; Lauckner et al., 2003; Iijima et al., 2010; Ando et al., 2016). The phosphorylated tau protein at this site is detected in preneurofibrillary tangles and is linked to an increased accumulation of seeding potency. Therefore, it is a starting point for tau abnormalities (Oba et al., 2020). On the other hand, phosphorylation in Ser262 alone does not participate in filament formation (Seubert et al., 1995; Schneider et al., 1999b). Recent studies showed that Δ280-tau and Δ187-tau mutants and phosphorylated K18Δ280 initially formed tangled dimers and tau protein oligomers (Barghorn et al., 2000; Nieznanska et al., 2021). So, we used Δ280-tau as a positive control and compared the results to one and three phosphorylation sites because the presence of P-Ser262 is important for tau protein dissociation from the microtubule, although it may not be directly involved in PHF formation.

We used the following methods to determine the effect of this phosphorylation: first, phosphorylation was performed on Ser262 in the sequence 254–290 of tau protein and docked two phosphorylated segments. Also, Δ280-tau and non-phosphorylated tau protein were prepared as the positive control and the control, respectively. After that, MD simulation was run for the two docked segments with phosphorylation of Ser262, Ser285, and Ser289 and positive control and control during 200 ns. Results showed that two segments that were just phosphorylated in Ser262 form an unstable complex with variable structures. In comparison, two segments with three phosphorylated sites formed compact and stable structures similar to positive control during simulations.

## Methods

### Molecular Docking

At first, the 3D structure of microtubule-associated tau protein (seq 254–290) was obtained from RCSB with ID: 5N5A, and phosphorylation was performed by CHARMM GUI. The segment was phosphorylated in two forms, including only phosphorylation on Ser262 and another on Ser626, Ser285, and Ser289. We used the non-phosphorylated complex to control and a mutated form of tau protein (Δ280-tau leads to the formation of PHF) to positive control. Then, complexes of these segments were prepared for MD simulation. Two segments, both phosphorylated at the Ser262 position, were docked in phosphorylated complexes. The second docked complex was performed for two phosphorylated segments at Ser626, Ser285, and Ser289. The control complex was prepared by docking two non-phosphorylated segments. To make a positive control, Δ280-tau was modeled using the Modeller program available in the Chimera visualization tool, and then the two mutant segments were docked. We used Zdock, HADDOCK, Cluspro, and Hawkdock web servers for docking segments, preparing complexes, finding binding sites, and binding free energy for each complex. The best-predicted structure was then selected from each tool based on the score. Finally, the Hawkdock program selected the best prediction based on the free binding energy obtained from the web servers (Yan et al., 2020).

### Molecular Dynamics Simulation

All-atom MD simulations of phosphorylated forms of tau protein were studied using GROMACS (version 2018.1) with CHARMM 36 force field. A TIP3P water model was used for the preparation of systems, and physiological concentrations of chloride and sodium (concentration of NaCl in cells is 0.15 M, which is considered a physiological concentration) were added to neutralize the systems (Callahan and Roux, 2018; Zavadlav et al., 2018; Alipour et al., 2022). The complexes are placed at least 1.0 nm in the center of the cubic box from the box edges. Furthermore, energy minimization was performed using the steepest descent algorithm with a maximum force <1,000.0 kJ/mol/nm to ensure that the system had no improper conformation. Then, equilibrium was done in a two-step, first by NVT and then NPT ensembles. A V-rescale thermostat was used to couple temperature at 310 K and a Parrinello–Rahman barostat for coupling pressure around 1 bar (Cino et al., 2012; Pachler et al., 2019). Finally, MD simulations were run for 200 ns in 2 fs and repeated three times (*n* = 3) for each complex (Supplementary Figs S1, S2). Periodic boundary conditions and saving energies in trajectory every 1 ps and constrain by the LINCS algorithm were set for runs too.

### Binding Energy and Dissociation Constant Calculations

The complexes’ binding energy components were calculated using the molecular mechanics Poisson–Boltzmann surface area method (MM-PBSA) by g_mmpbsa in GROMACS. Moreover, the dissociation constant (Kd) was calculated with the formula (Iqbal et al., 2010) where R is the gas constant, T is the temperature in Kelvin, and ∆G is the binding energy. Kd is an important parameter to indicate the degree of affinity between two segments (Luo and Sharp, 2002).

ΔG=−RTlnKd . (1)

## Results and Discussion

### Stability and Flexibility

**FIGURE 1:**
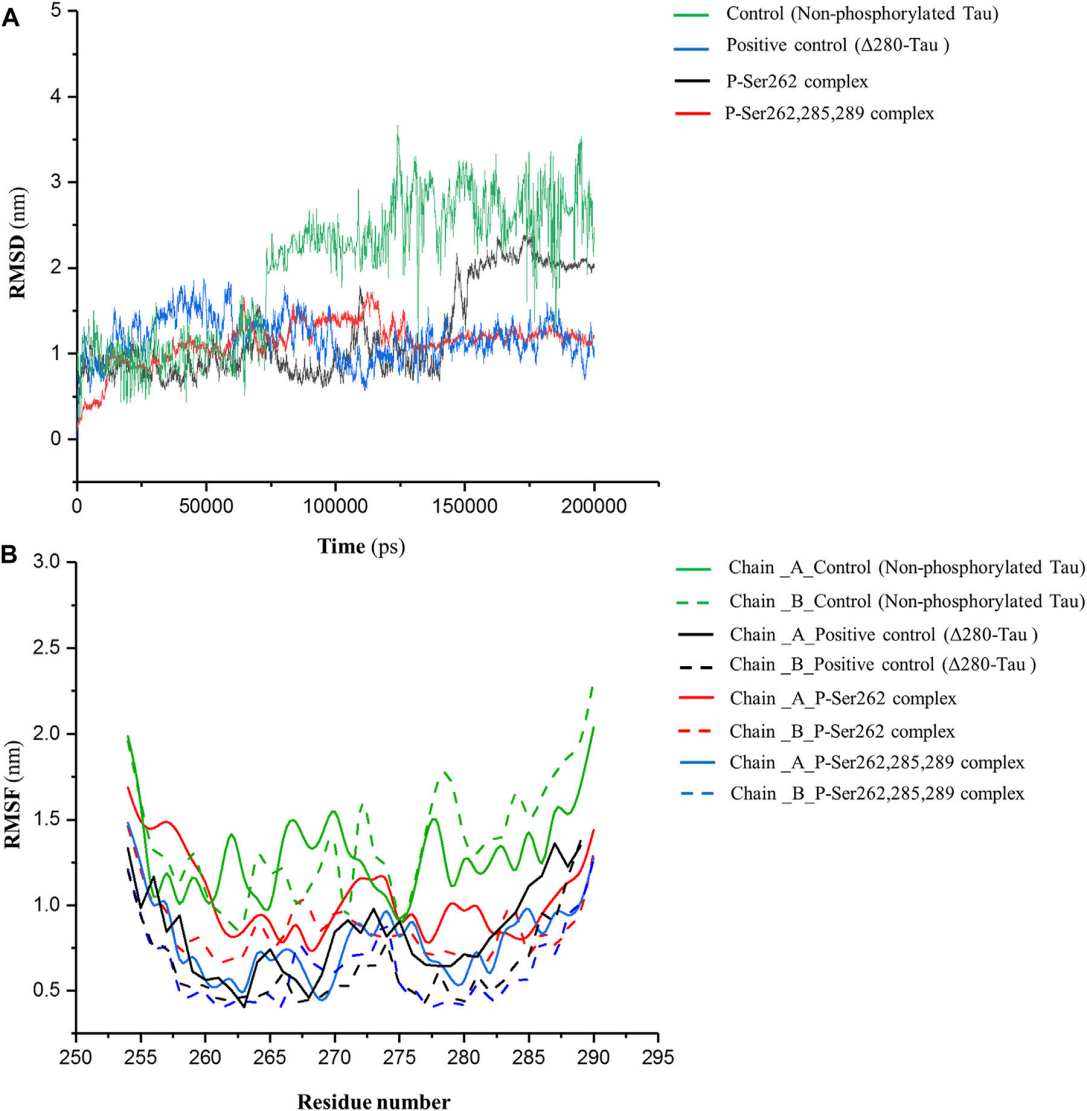
RMSD of backbone and RMSF profile for the control (non-phosphorylated tau), positive control (Δ280-tau), and the complexes with one (P-Ser262) and three (P-Ser262, 285, and 289) phosphorylation sites during 200 ns simulations. **(A)** RMSD plots representing the stability of the complexes and **(B)** RMSF of residues show each strand’s flexibility.

MD simulation was employed for the complexes, including two docked similar segments that are phosphorylated in the Ser262 situation, and another one, the phosphorylated segments in Ser262, Ser285, and Ser289. Also, we used the non-phosphorylated and Δ280-tau complexes for the control and the positive control, respectively. As shown in Figure 1A, root mean square deviation (RMSD) fluctuated sharply in the control complex and the complex with one phosphorylated site (P-Ser262) so that the RMSD value in these complexes increased to near 3 and 2.5 nm, respectively, but the RMSD of the positive control and the complex with three phosphorylated sites (P-Ser262, 285, and 289) were stabled around 1 nm. This means more structural changes occurred in the P-Ser262 complex when compared to the P-Ser262, 285, 289 complex during 200 ns simulations. Moreover, results indicated that the root mean square fluctuation (RMSF) value of strands with one phosphorylated site and the control was higher than strands with three phosphorylated sites and positive control. Indeed, strands’ flexibility and structural changes have decreased with the increase in the number of phosphorylated sites. (Figure 1B).

### Analysis of Interactions

**FIGURE 2:**
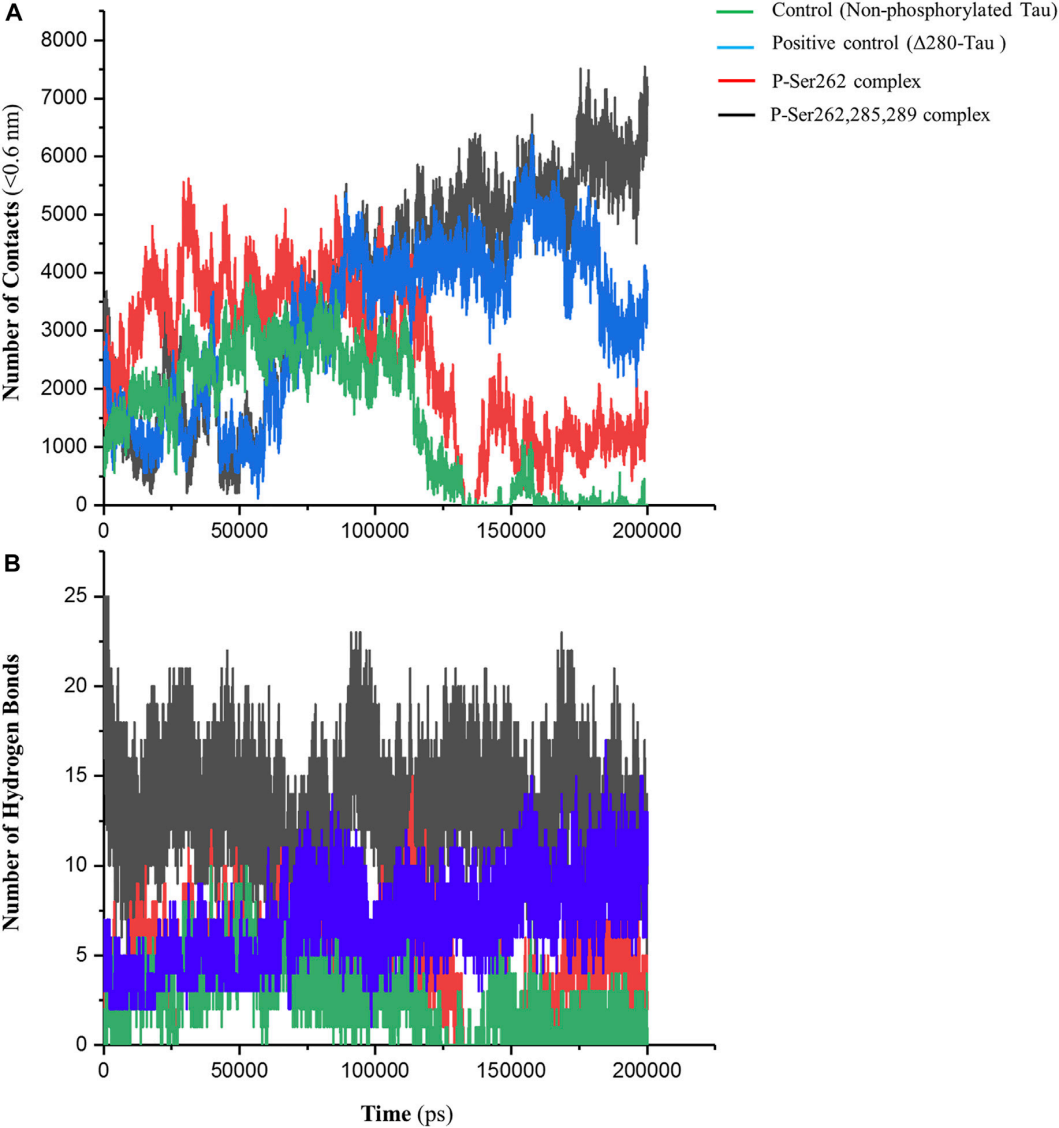
Number of contacts (NC) and number of hydrogen bonds (NHB) between two segments in the control (non-phosphorylated tau), positive control (Δ280-tau), and the complexes with one (P-Ser262) and three (P-Ser262, 285, and 289) phosphorylated sites during 200 ns simulations. **(A)** NC was calculated at a distance of less than 0.6 nm, which decreased in the P-Ser262 compared to P-Ser262, 285, and 289. **(B)** NHB in P-Ser262 is less than in P-Ser262, 285, and 289.

The number of contacts (NC) is an important parameter to analyze atomistic-resolved descriptions of the interactions occurring within and between the researched molecules. Indeed, NC can measure the binding affinity of one molecule to another (Liu et al., 2007). So, NC between two segments was calculated in control, positive control, and P-Ser262 and P-Ser262, 285, 289 complexes at a distance of less than 0.6 nm (r < 0.6 nm). This distance can be a criterion for the potential of atom-to-atom interactions such as hydrogen bonds, electrostatic interactions, and hydrophobic interactions. As shown in Figure 2A, the NC of the control and P-Ser262 complexes decreased when compared to the positive control and the P-Ser262, 285, 289 complexes during 200 ns simulations. Moreover, NC of the P-Ser262, 285, 289 complex shows an increasing trend when compared to other complexes. These results indicated that NC between two segments in the control and the P-Ser262 complex reached zero, meaning the two segments were separated at some time points.

On the other hand, the NC of the P-Ser262 complex was more than that of control. Moreover, as another parameter to satisfy binding affinity, the number of hydrogen bonds (NHB) was calculated for each complex during simulations. Results showed that NHB in control and P-Ser262 complexes was less than that in positive control and P-Ser262, 285, 289 complex, so their average was about 4, 5, 14, and 16, respectively (Figure 2B). In general, the results showed that the interactions of the P-Ser262 complex were very similar to the control, but they increased significantly with increasing the number of phosphorylated sites to three. Indeed, as NC and NHB increase in P-Ser262, 285, 289 complex, the possibility of stable dimers increases, which is the first step in PHF formation (Luna-Viramontes et al., 2020).

### Interaction Energy Analysis

**FIGURE 3:**
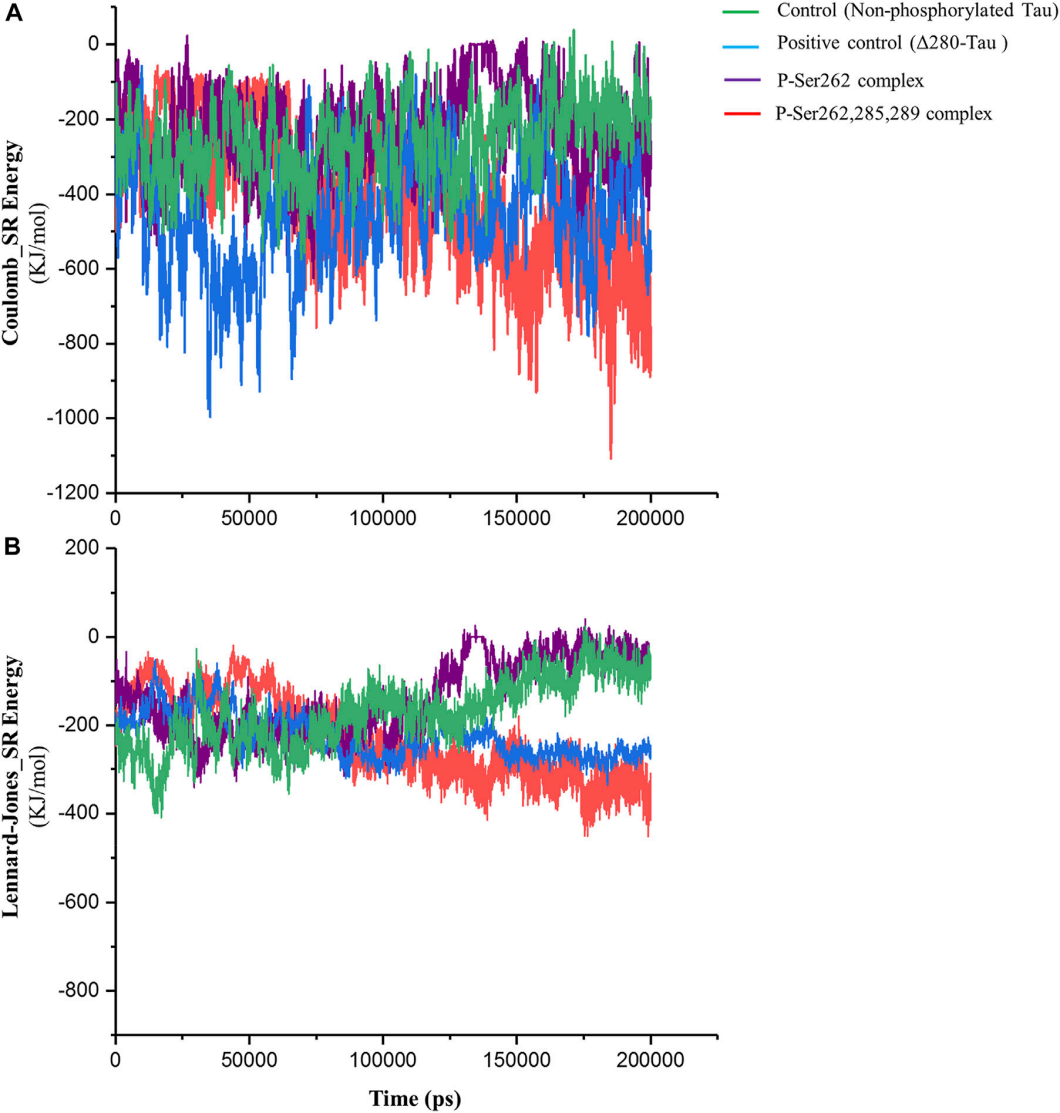
Short-range Coulomb and Lennard-Jones energies between two segments in the control (non-phosphorylated tau), positive control (Δ280-tau), and the complexes with one (P-Ser262) and three (P-Ser262, 285, and 289) phosphorylated sites during 200 ns simulations. **(A)** Coulomb and **(B)** Lennard-Jones energies are more negative in P-Ser262, 285, and 289 compared to P-Ser262.

Short-range Lennard-Jones and Coulomb energies describe interaction energy between two non-bonding atoms or molecules (Van Lipzig et al., 2004). So, these energies were calculated between two segments in control, positive control, and P-Ser262 and P-Ser262, 285, 289 complexes. As shown in Figure 3A, Coulomb energy of P-Ser262, 285, 289 complex becomes more negative as well as positive control than P-Ser262 during simulations, so the average value of these energies in P-Ser262, 285, and 289 and P-Ser262 was about −516.63 and −230.99 kJ/mol, respectively. Moreover, the Coulomb energy of the control and P-Ser262 complexes was zero at some time points, which means the complexes were separated into two segments during the simulations. The obtained results in Lennard-Jones energy were similar to those in Coulomb energy, and the average values of these energies in P-Ser262 and of P-Ser262, 285, 289 complexes were −156.81 and −254.63 kJ/mol, respectively. (Figure 3B). The more negative values of these energies in P-Ser262, 285, and 289 than in P-Ser262 signify that two segments were tightly bound together and made a stable dimer as an important step in PHF formation.

### Radius of Gyration

**FIGURE 4:**
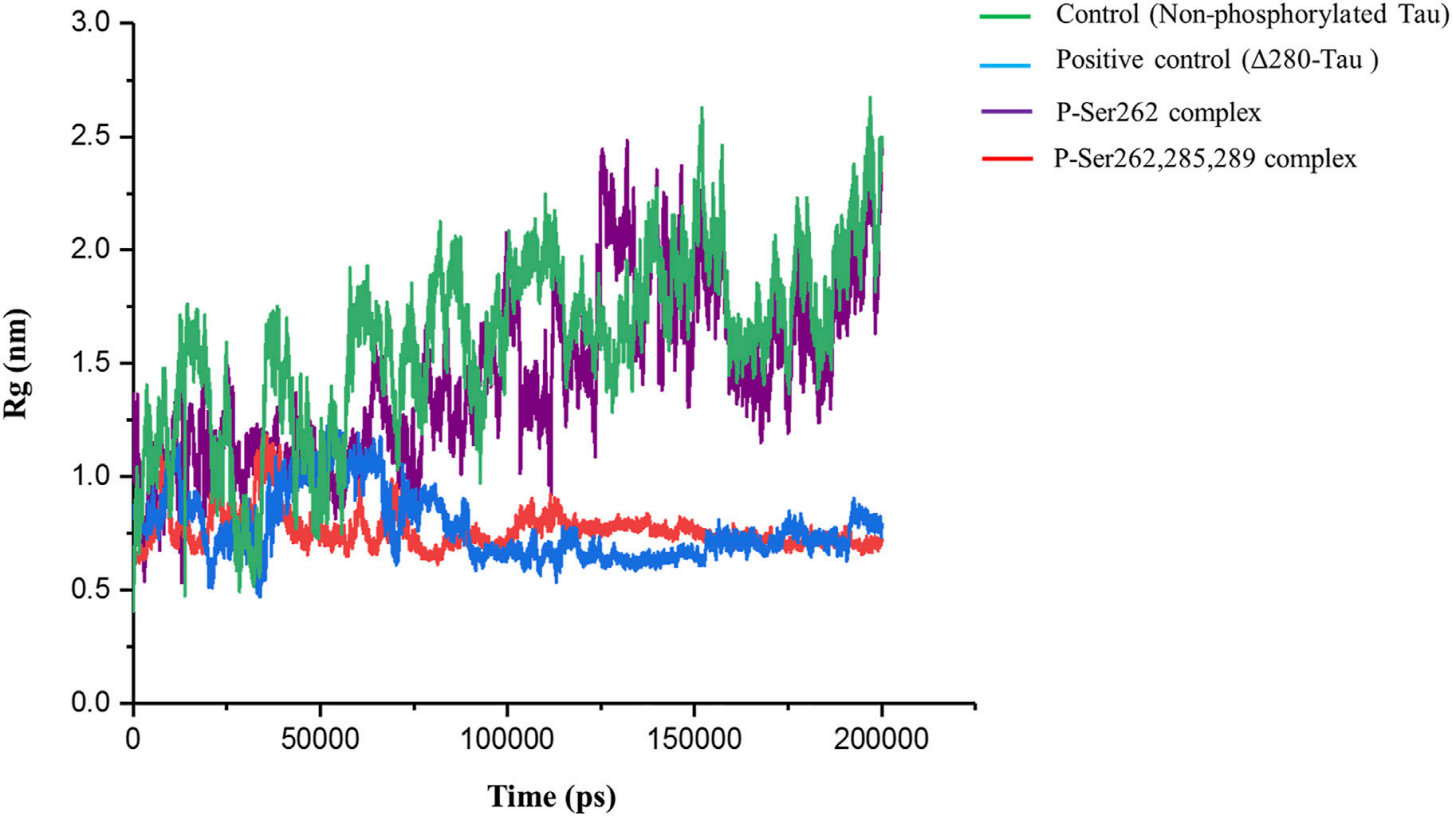
Change in the radius of gyration (Rg) of the two docked segments in the control (non-phosphorylated tau), positive control (Δ280-tau), and the complexes with one (P-Ser262) and three (P-Ser262, 285, and 289) phosphorylated sites during 200 ns simulations. Rg values were increased mainly in the P-Ser262 compared to the P-Ser262, 285, 289.

Radius of gyration (Rg) is a standard parameter to measure molecular size. It is the RMSD to the center of mass in the atom, molecule, peptide, protein, etc. Using this parameter, the compaction rate of one molecule can be compared with others (He and Niemeyer, 2003). So, Rg was calculated to find compaction of the complexes including the control, positive control, and P-Ser262 and P-Ser262, 285, 289 complexes during 200 ns simulations. Results indicated that Rg of the control and the P-Ser262 complex fluctuated. However, Rg values in the positive control and P-Ser262, 285, 289 complexes fluctuated up to 115 ns and stabilized around 0.75 nm. Also, Rg values were increased mainly in the control and P-Ser262 complexes when compared to the positive control and P-Ser262, 285, 289 complexes (Figure 4). Increasing the Rg value means that the structure compaction declined and two segments were separated during the simulation.

### Principal Component Analysis

**FIGURE 5:**
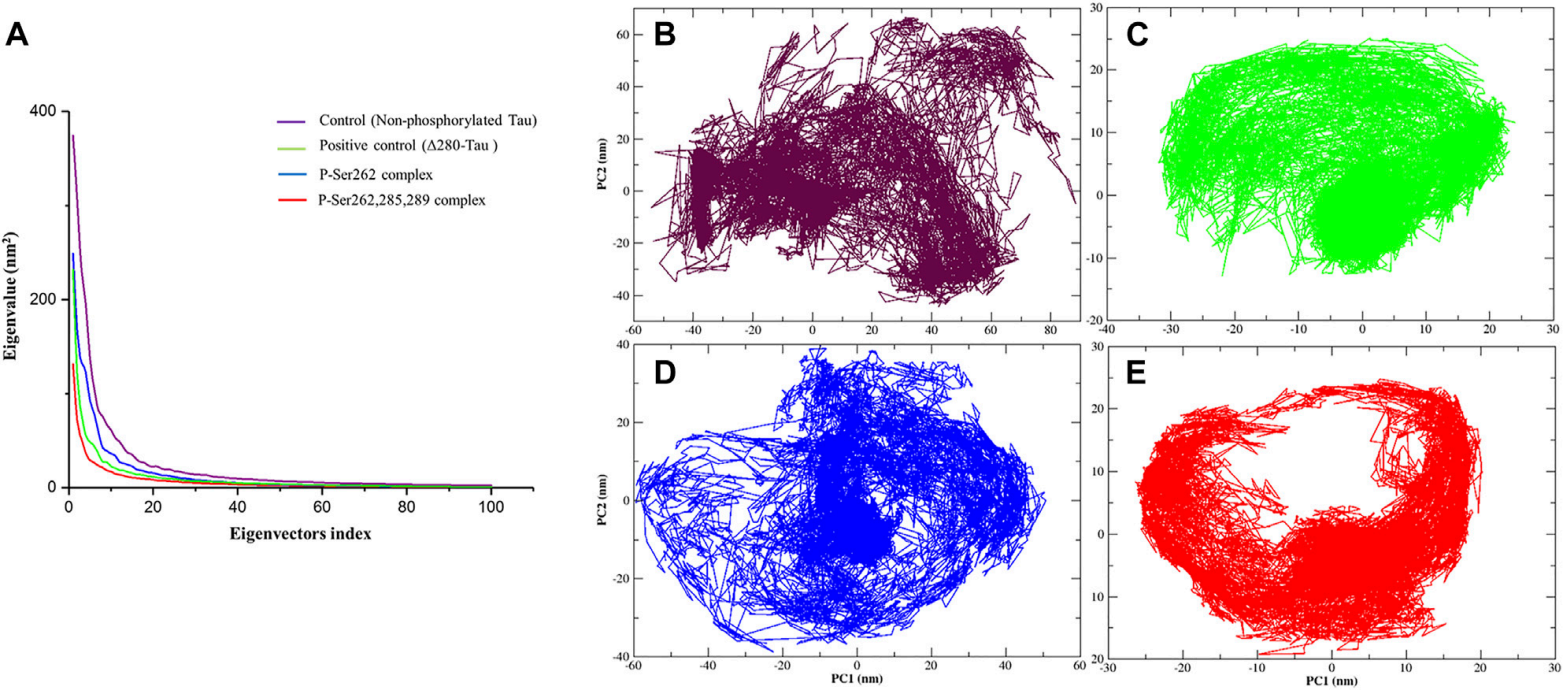
Principal component analysis (PCA) of the Cα atomic fluctuations in the control (non-phosphorylated tau), positive control (Δ280-tau), and the complexes with one (P-Ser262) and three (P-Ser262, 285, and 289) phosphorylated sites during 200 ns simulations. **(A)** Eigenvalues were plotted against the eigenvectors. Projections of the trajectory of **(B)** control and **(D)** P-Ser262 showed more changes compared to **(C)** positive control and **(E)** P-Ser262, 285, 289.

Principal component analysis (PCA) is a linear transform to analyze the motion of a molecule using a covariance matrix, which is constructed from atomic displacements (Cartesian coordinates) in each conformation during simulation. Eigenvectors are obtained from diagonalization of covariance matrix used to explain molecule motions. Lower eigenvectors signify lower motion and high compactness of a molecule during simulation (David and Jacobs, 2014; Fatima et al., 2020). So PCA was calculated for the control, positive control, and P-Ser262 and P-Ser262, 285, 289 complexes from the Cα covariance matrix during 200 ns simulations. Obtained results of plotting eigenvalues against the eigenvectors indicated that 90% of the complexes’ motion was covered by the first 10 eigenvectors and reached their equilibrium in the first 20 eigenvectors (Figure 5A). Moreover, eigenvectors of the control and P-Ser262 complexes were higher than those of the positive control and P-Ser262, 285, 289 complexes, covering the areas on PC1 and PC2 that were between −60 and 80, −60 and 60 in the control and −60 and 60, −40 and 40 in the P-Ser262 complex, respectively, while in both PC1 and PC2 were between −20 and 20 in the positive control and the P-Ser262, 285, 289 complexes (Figures 5B–E). Indeed, PCA results generally suggested that various structural changes occurred in the control and P-Ser262 complexes that caused P-Ser262, 285, 289 complexes to be more stable than P-Ser262.

### Gibbs Free Energy Landscape Analysis

**FIGURE 6:**
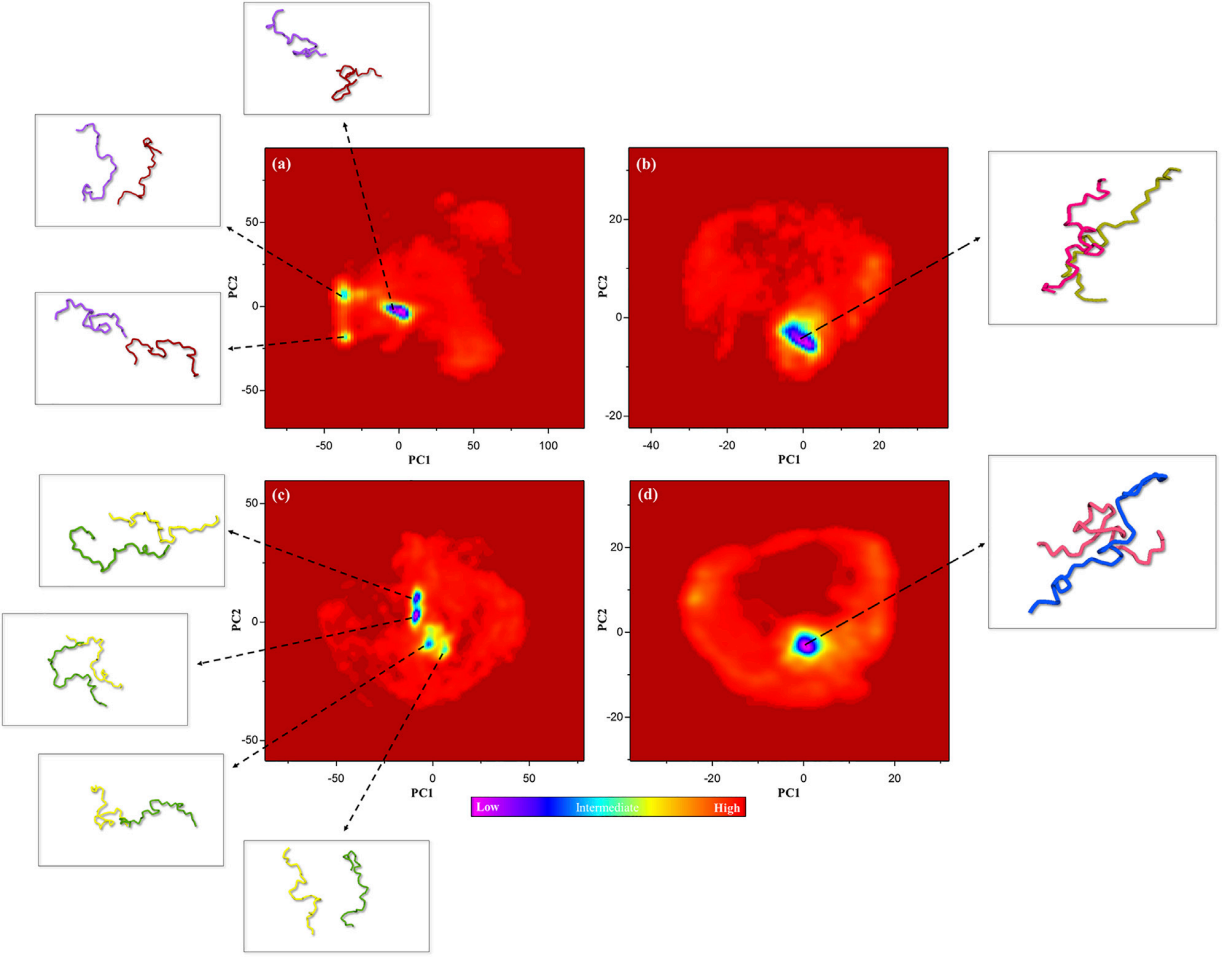
Free energy landscape (FEL) of the control (non-phosphorylated tau), positive control (Δ280-tau), and the complexes with one (P-Ser262) and three (P-Ser262, 285, and 289) phosphorylated sites on principal components 1 and 2 during 200 ns simulations. Multiple minimum-energy clusters were observed in **(A)** the control and **(C)** P-Ser262, but one cluster was observed in **(B)** the positive control and **(D)** P-Ser262, 285, 289.

Gibbs’s free energy landscape (FEL) is an important measure of molecule stability that allows finding the native state structures by the conformational sampling method. Native states are stable structures with minimum FEL during simulation (Borkotoky and Murali, 2018; Amir et al., 2019). Thus, FELs of the control, positive control, and P-Ser262 and P-Ser262, 285, 289 complexes were calculated to identify the conformational states using the first two principal components. Results indicated multiple minimum-energy clusters in the control and P-Ser262 complexes that depicted the structural transition from one structure to different active conformational states, while there was one minimum-energy cluster in the positive control and P-Ser262, 285, 289 complexes. Indeed, the P-Ser262, 285, 289 complex was the most stable compared to the control and P-Ser262 complex, so the two segments were wholly separated in these complexes at some time points. In contrast, a stable minimum-energy dimmer is formed in P-Ser262, 285, 289 with a similar positive control (Figure 6).

### Binding Energy and Dissociation Constant

Binding energy (ΔG) and dissociation constant (Kd) are important parameters to estimate the stability and affinity of two segments (Wang et al., 2017). So, these parameters were calculated for the control, positive control, and P-Ser262 and P-Ser262, 285, 289 complexes during 200 ns simulations. Results showed that ΔG of the positive control and P-Ser262, 285, 289 complexes were more negative than the control and P-Ser262 complexes, and their values were −76.36 kJ/mol, -80.26 kJ/mol, −3.69 kJ/mol, and −6.29 kJ/mol, respectively. Also, Kd in the positive control and P-Ser262, 285, 289 were lower than that in the control and P-Ser262 (Table 1). So, as a result, two segments in the P-Ser262, 285, 289 complex were tightly bound together compared to the P-Ser262 complex and did not separate easily.

**TABLE 1:** Binding energy (∆G) and dissociation constant (Kd) values for the control (non-phosphorylated tau), positive control (Δ280-tau), and the complexes with one (P-Ser262) and three (P-Ser262, 285, and 289) phosphorylation sites during 200 ns simulations.

Complex	ΔG (KJ/mol)	Kd (M) at 37.0 (˚C)
Control	−3.69	2.3 × 10^−2^
Positive control	−76.36	1.35 × 10^−13^
P-Ser262	−6.29	8.7 × 10^−2^
P-Ser262, 285, 289	−80.26	2.99 × 10^−14^

In conclusion, increasing tau phosphorylation occurs in the early stage of AD. Indeed, hyperphosphorylation of tau protein decreases its capacity to bind to microtubules, leading to microtubule destabilization and disruption of the axons and dendrites, the formation of neurofibrillary tangles, and finally, neuronal death. Studies have shown that phosphorylation of Ser262 is an important marker to distinguish AD, and also, several motifs are hotspots for phosphorylation (Majd et al., 2017). Our results show that the P-Ser262 complex is much more unstable than a P-Ser262, 285, 289 complex, and there are many structural fluctuations. Moreover, two segments in the P-Ser262 complex are separated so that the number of contacts, number of hydrogen bonds, and interaction energy become zero at some time points during simulation. So, Rg of the P-Ser262 complex dramatically increased when compared to the P-Ser262, 285, 289 complex. Indeed, the results obtained from the MD simulation of P-Ser262 are very similar to the non-phosphorylated complex (control).

Since the two segments are not tightly bonded in the P-Ser262 complex, they have a higher dynamic than the P-Ser262, 285, 289 complex. On the other hand, the high structural dynamics of the P-Ser262 complex increases the likelihood of diverse structures in it, so that there are several clusters in the P-Ser262 than in the P-Ser262, 285, 289 complex. Evidence implies that small aggregated protein intermediates, which may form soluble oligomers before forming highly structured fibrils, are the principal toxic species involved in starting neurodegenerative disorders (Vieira et al., 2007; Peterson et al., 2008; Pavlova et al., 2016). In other words, the formation of intertwined dimers and oligomers is the first step in forming PHFs and finally the fibrils. PHF structures can be assembled from a different number of these subunits (Barghorn et al., 2000; Nieznanska et al., 2021). On the other hand, our results showed that phosphorylation at these three sites leads to an intertwined dimer that may create more complex structures such as PHFs. Generally, our study shows that two segments only phosphorylated in Ser262 form an unstable complex with variable structure than two segments with three phosphorylated sites.

## Data Availability

The raw data supporting the conclusion of this article will be made available by the authors, without undue reservation.
