# Supplementary material for: Structural Analysis and Conformational Dynamics of Short Helical Hyperphosphorylated Segments of Tau Protein (Sequence 254–290) in Alzheimer’s Disease: A Molecular Dynamics Simulation Study
Source: Front Mol Biosci. 2022 Aug 8;9:884705. doi: 10.3389/fmolb.2022.884705 (PMC9393928; doi:10.3389/fmolb.2022.884705)

Supplementary information for:

Structural analysis and conformational dynamics of short helical hyperphosphorylated segments of Tau protein (sequence 254-290) in Alzheimer's disease: A molecular dynamics simulation study

Mozhgan Alipour[1,2], Mahsa Motavaf[1], Parviz Abdolmaleki[2], Alireza Zali[1], Farzad Ashrafi[1], Saeid Safari[1,*], Behnam Hajipour-Verdom[1,2,*].

[1] *Functional Neurosurgery Research Center, Shohada Tajrish Comprehensive Neurosurgical Center of Excellence, Shahid Beheshti University of Medical Sciences, Tehran, Iran.*

[2] *Department of Biophysics, Faculty of Biological Sciences, Tarbiat Modares University, Tehran, 14115-154, Iran.*

***Corresponding Authors:**

1- Saeid Safari, Tel.: +98 21 2272 4211. E-mail address: safari.s@sbmu.ac.ir

2- Behnam Hajipour-Verdom, Tel.: +98 21 8288 4467. E-mail address: b.hajipour@modares.ac.ir

Supplementary Figures and Legends

Supplementary Figure S1. Root mean square deviation (RMSD) of backbone for the **(a)** control (non-phosphorylated tau), **(b)** positive control (Δ280-tau), and the complexes with **(c)** one (P-Ser262) and **(d)** three (P-Ser262, 285, 289) phosphorylation sites, during 200 ns simulations that are repeated three times (n = 3). RMSD plots represent the stability of the complexes.

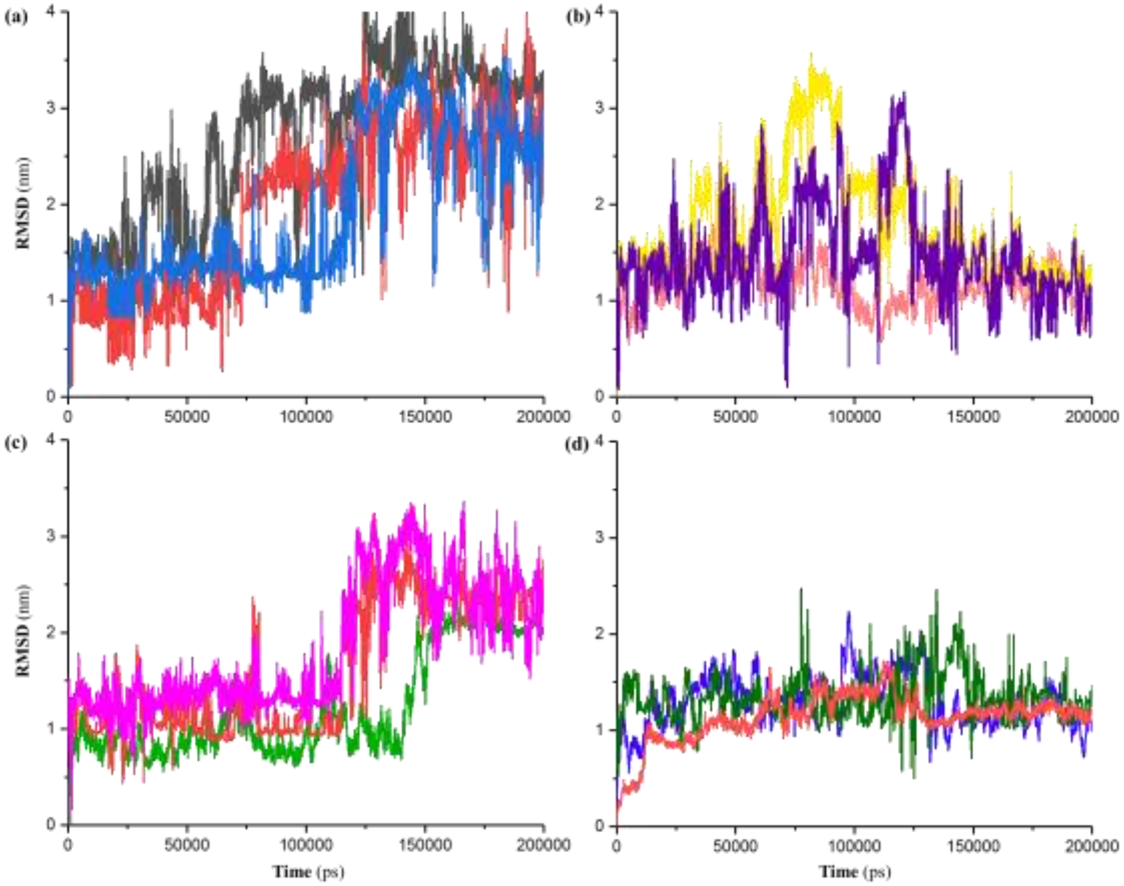

Supplementary Figure S2. Change in the radius of gyration (Rg) of the two docked segments in **(a)** the control (non-phosphorylated tau), **(b)** positive control (Δ280-tau), and the complexes with **(c)** one (P-Ser262) and **(d)** three (P-Ser262, 285, 289) phosphorylated sites during 200 ns simulations that are repeated three times (n = 3). The Rg is a standard parameter for examining the compaction of a structure that can be used to compare the stability of the structures.

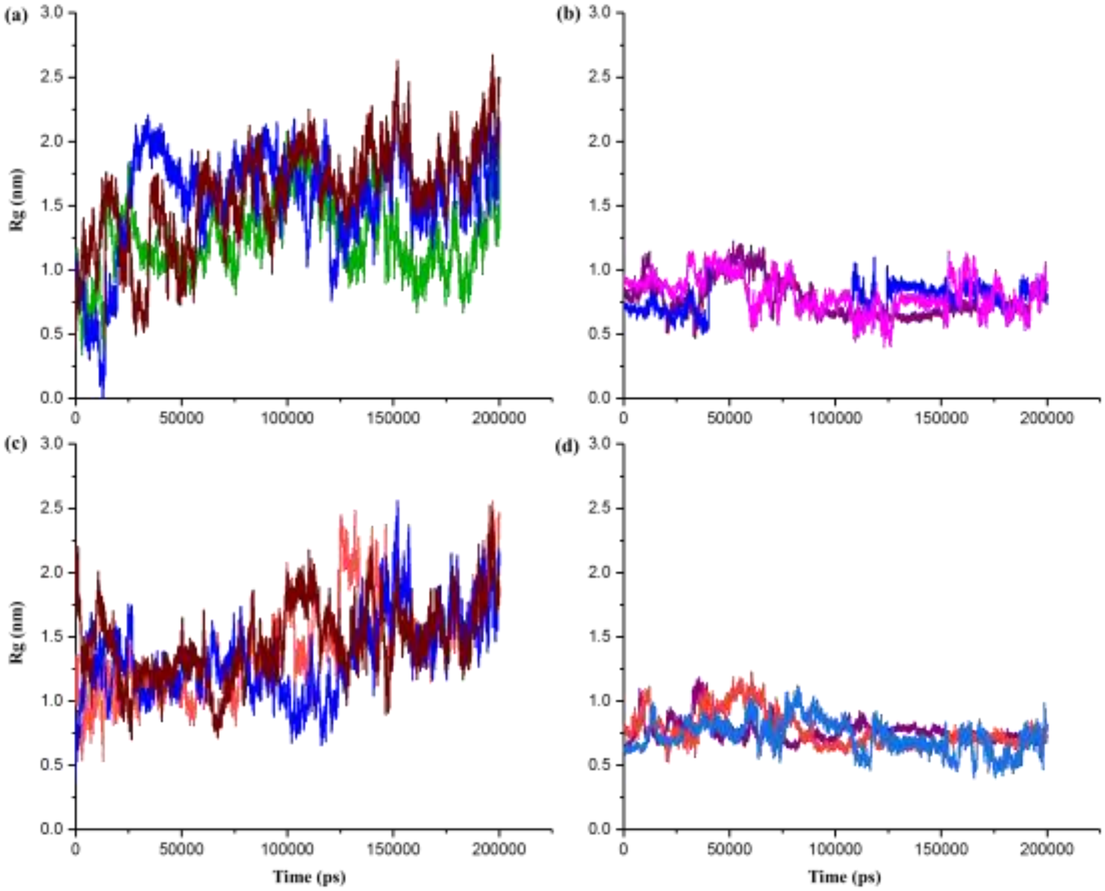

Supplement: Supplementary file 1 [file DataSheet1.pdf]
